# Characterization of a novel chitinolytic *Serratia marcescens* strain TC-1 with broad insecticidal spectrum

**DOI:** 10.1186/s13568-022-01442-6

**Published:** 2022-07-30

**Authors:** Aili Tao, Tan Wang, Fahu Pang, Xueling Zheng, Camilo Ayra-Pardo, Siliang Huang, Ruxin Xu, Fengqin Liu, Jiakang Li, Yibin Wei, Zhiqing Wang, Qiuhong Niu, Dandan Li

**Affiliations:** School of Life Science and Agricultural Engineering, Nanyang Normal University, Nanyang, 473061 China

**Keywords:** *Serratia marcesecens*, Insect pathogen, *Anomala corpulenta*, White grub, Biological control, Chitinase, Response surface methodology

## Abstract

The Gram-negative rod-shaped bacterium *Serratia marcescens* is an opportunistic pathogen of many organisms, including insects. We report the identification and optimal in vitro chitinase production conditions of a novel chitinolytic *S. marcescens* strain TC-1 isolated from a naturally infected white grub (*Anomala corpulenta*) collected from a peanut field at Nanyang city, Henan province, China. Strain identification was conducted by morphological, physiological, biochemical and molecular analyses. The amplified 16S rRNA gene of TC-1 showed a similarity greater than 99% with multiple strains of *S. marcescens*. Based on Neighbor-joining phylogenetic tree analysis of bacterial 16S rRNA gene sequences, TC-1 formed a clade with *S. marcescens*, clearly separated from other *Serratia* spp. The strain TC-1 showed larvicidal activities against five insect species (*A. corpulenta*, *Plutella xylostella*, *Spodoptera exigua*, *Helicoverpa armigera*, *Bombyx mori*) and the nematode *Caenorhabditis elegans*, but not against *S. litura*. The operating parameters of chitinase production by TC-1 were optimized by response surface methodology using a three-factor, three-level Box-Behnken experimental design. The effects of three independent variables i.e. colloidal chitin concentration (7–13 g l^−1^), incubation time (24–72 h) and incubation temperature (24–32 °C) on chitinase production by TC-1 were investigated. A regression model was proposed to correlate the independent variables for an optimal chitinase activity predicted as 20.946 U ml^−1^, using a combination of colloidal chitin concentration, incubation time and incubation temperature of 9.06 g l^−1^, 63.83 h and 28.12 °C, respectively. The latter agreed well with a mean chitinase activity of 20.761 ± 0.102 U ml^−1^ measured in the culture supernatants of TC-1 grown under similar conditions with a colloidal chitin concentration, incubation time and incubation temperature of 9 g l^−1^, 64 h and 28 °C, respectively. Our study revealed the *S. marcescens* strain TC-1 with potential as a biocontrol agent of insect pests and nematodes and demonstrated the proposed regression model's potential to guide chitinase production by this strain.

**Supplementary Information:**

The online version contains supplementary material available at 10.1186/s13568-022-01442-6.

## Key points

- *A new Serratia marcescens strain TC -1 showed high insecticidal activity against several phytophagous insect species and nematocidal activity against Caenorhabditis elegans.*
- *The larvae of S. litura differed in their susceptibility to TC-1 compared to other Lepidoptera insect species.*
- *A regression model correlated well with the variables (chitin concentration, time and temperature) for high chitinase production by TC -1.*

## Introduction

*Serratia marcescens* is a Gram-negative bacillus that occurs naturally in soil, water, foodstuff and animals (Hejazi and Falkiner 1997). The bacterium produces a red pigment, named prodigiosin, though this ability is absent in some strains isolated from humans (Gargallo et al. 1987; Grimont and Grimont 1978).

Considerable diversity has been found among *S. marcescens* populations. Many strains of the bacterium are known to be saprophytic in soil (Nawani and Kapadnis 2001) or endophytic in plants (Gyaneshwar 2001), but some are notorious for causing diseases on plants (Bruton et al. 1995, 2003; Lukezic et al. 1982; Sears et al. 1975; Wang et al. 2015) and animals (Quesenberry and Short 1983) as well as on humans (Whalen 1970; Zipper et al. 1996). Several studies have been focused on the beneficial functions of some *S. marcescens* strains, such as biodegradation and bioremediation potential of environmental pollutants (Abo-Amer 2011; Cycoń et al. 2012); as a bio-collector for hematite flotation (Yang et al. 2014a, b); plant-growth-promoting potential (George et al. 2013; Lavania et al. 2006; Selvakumar et al. 2008); the biocontrol of insect pests (Deng et al. 2008; Fu et al. 2021; King et al. 1975; Podgwaite and Cosenza 1976; Qi et al. 2004; Sikorowski et al. 2001; Wang et al. 2010; Yang et al. 2014a, b; Zhao et al. 2017; Zhang et al. 2011, 2021), plant pathogens (Ahmed 2010; Dong et al. 2016; Feng et al. 2018; Someya et al. 2001), plant diseases (Ordentlich et al. 1987 and 1988; Someya et al. 2000; Wei et al. 1996) and weeds (Yang et al. 2015a, b; Li et al. 2021).

The virulence of *S. marcescens* on both insects and fungi is due in part to its chitinase enzymes, which hydrolyze chitin—the second most abundant natural biopolymer after cellulose that constitutes the major structural component of certain rigid structures in invertebrates (e.g. the insects' exoskeleton) and the cell walls of fungi (Shahidi et al. 1999; Nawani and Kapadnis 2001; Merzendorfer and Zimoch 2003). Chitin metabolism is an essential life sustaining activity of phytophagous insects, phytopathogenic fungi and parasitic nematodes, which are the major limiting factors of the agricultural production system (Subbanna et al. 2018). For this reason, the breakdown of chitin or inhibition of chitin metabolism can lead to the death of these agricultural pests. Therefore, isolation and characterization of chitinolytic *S. marcescens* strains are considered crucial for the development of efficient biocontrol agents against insect pests (Chen et al. 2001; Li 1982; Liu et al. 2019; Lysenko 1976; Parani et al. 2011; Sezen et al. 2001; Yang et al. 2015a, b; Yin et al. 2004) and plant pathogenic fungi (Babashpour et al. 2012; Gutiérrez-Román et al. 2012; Kobayashi et al. 1995; Moon et al. 2017; Oppenheim and Chet 1992; Parani et al. 2011; Someya et al. 2005). Chitinase production by a bacterium is mainly influenced by the culture conditions (Gutiérrez-Román et al. 2012), which must be optimized for the individual bacterial strains.

The metallic green beetle *Anomala corpulenta* Motschulsky (Coleoptera: Scarabaeidae: Rutelinae) is an economically important insect pest throughout Asia including China. The larvae (white grubs) and adults of this insect pest commonly infest the underground and aboveground parts of multiple plant species, respectively. The adults feed preferably on the leaves of apple, pear, grape, peanut, soybean, poplar and elm (Ji et al. 2017; Li et al. 2009), resulting in significant damage to agriculture, forestry and urban greening in the severe cases. The primary control methods for *A. corpulenta* rely heavily on conventional pesticide sprays (Gong et al. 2016; Ji et al. 2017), which is a concern for the environment, biodiversity and human health. Microbe-based biopesticides can provide an economical, ecofriendly and sustainable approach to insect pest management.

In 2013, we isolated a new chitinolytic *S. marcescens* strain TC-1 from a naturally infected white grub (*A. corpulenta*) and found that its culture supernatants (CS) could cause the death of second- and third-instar larvae of *Spodoptera exigua* (Additional file 1: Table S1). Isoelectric point screening tests of proteins in TC-1’s CS revealed that the protein with the highest chitinolytic activity precipitated at pH 6.7 (Additional file 1: Tables S2 and S3). Furthermore, we used the protein precipitated at pH 6.7 as a crude chitinase and confirmed its larvicidal activity against *S. exigua* (Additional file 1: Table S4). In the present work, we aimed to identify and characterize the strain TC-1 by evaluating its pathogenicity against larvae of six phytophagous insect species and one nematode, and optimizing its chitinase production conditions using response surface methodology (RSM).

## Materials and methods

### Insects and nematode

The following insect larvae were used in this study: *A. corpulenta* (white grub), *Bombyx mori* (silkworm), *Helicoverpa armigera* (cotton bollworm), *Plutella xylostella* (diamondback moth), *S. exigua* (beet armyworm) and *S. litura* (common cutworm). The larvae of *A. corpulenta* and *B. mori* were reared at our university on potato slices and mulberry leaves, respectively, at 25 °C ± 1 °C and a photoperiod of 16/8 h (light/dark). The insects of *H. armigera*, *P. xylostella*, *S. exigua* and *S. litura* in the second and third larval instars and their artificial diets were purchased from Henan Baiyun Industrial Co., Ltd. (China). A population of nematode *Caenorhabditis elegans* was maintained in the laboratory of microbiology of the Nanyang Normal University using the standard technique (Stiernagle 2005).

### Bacterial strain isolation

In August 2013, a naturally infected white grub (*A. corpulenta*) was collected from a peanut field located at the western campus of Nanyang Normal University (112° 28′ 44"N, 32°58′ 34"E, 131 m above sea level). The insect body was 1.5 cm in length and showed clear symptoms of disease with a pale violet red color. Surface disinfection was carried out as follows: The affected insect was dipped in 70% ethanol for 10 s, followed by 4 min in 0.1% HgCl_2_, and five rinses with sterile water. The surface-disinfected insect was homogenized in five ml of sterilized water with a sterilized pestle to produce a bacterial suspension that was plated (200 μl per plate) on beef-peptone-yeast-dextrose agar (BPYDA: beef extract, 3 g l^−1^; peptone, 5 g l^−1^; yeast extract, 1 g l^−1^; dextrose, 10 g l^−1^; agar, 15 g l^−1^; pH 7.0). The plates were incubated at 28 °C for 24 h to obtain bacterial colonies that were further purified in three rounds of single-colony isolation on BPYDA. A representative strain (TC-1) was randomly selected and identified through morphological, physiological, biochemical, and molecular methods. The strain TC-1 was deposited at the China Center for Type Culture Collection (CCTCC) with serial number M2015634.

### Characterization and identification of the isolated strain

The morphological analysis was carried out by recording the phenotypic features of TC-1 colonies grown on BPYDA plates for 48 h at 28 °C. For the TC-1’s physiological and biochemical characterizations, 28 reactions i.e. oxidase, Gram-staining, phenylalanine deaminase, lysine decarboxylase, ornithine decarboxylase, DNase, arginine decarboxylase, urease, motility, productions of H_2_S and indole, acid production from glucose, Voges-Proskauer (V.-P.), methyl red, utilization of the carbon sources (mannitol, melibiose, sucrose, sorbitol, lactose, adonitol, xylose, raffinose, bile esculin, and L-arabinose), gelatin liquefaction, Simmons citrate, malonate, and reduction of nitrate, were performed using routine bacteriological methods (Buchanan and Gibbon 1984; Dong and Cai 2001).

For the molecular identification, the TC-1’s genomic DNA was extracted using a procedure previously described by Tao et al. (2014), and used for PCR of the 16S rRNA gene sequence with universal primers 27F (5´-AGAGTTTGATCATGGCTCAG-3´) and 1492R (5´-TACGGTTACCTTGTTACGACTT-3´) in a 50-μl reaction mixture (Sambrook and Russell 2001). The PCR reaction was run for 30 cycles of DNA denaturation for 60 s at 94 °C, annealing for 30 s at 53 °C, and extension for 60 s at 72 °C. The amplified product was visualized following electrophoresis in 1.0% agarose gels stained with GelRed (Biotium) and sent for bi-directional sequencing using primers 27F and 1492R. Sanger sequences were generated at Shanghai Bajun Biological Technology Co., Ltd. (China). Generated sequences were converted to Fasta format and compared with the bacterial 16S rRNA gene sequences deposited in GenBank using the algorithm BLAST (https://blast.ncbi.nlm.nih.gov/Blast.cgi). A Neighbor-joining phylogenetic tree of the TC-1's 16S rRNA sequence with other bacterial 16S rRNA gene sequences retrieved from the NCBI GenBank was reconstructed using the Molecular Evolutionary Genetics Analysis version 7.0 (MEGA7) under 1000 bootstrap replicates.

### Pathogenicity tests

The TC-1's virulence against six insect species (*A. corpulenta*, *B. mori*, *H. armigera*, *P. xylostella*, *S. exigua*, and *S. litura*) and the nematode *C. elegans* was investigated through bioassays. To prepare the inocula, a loopful of TC-1 colony grown on a BPYDA slant was inoculated into a BPYDB liquid medium (the same components as BPYDA except agar) and grown overnight at 28 °C with vigorous shaking (180 rpm). One ml of this culture was then used to inoculate 250-ml BPYDB and allowed to grow in a rotary incubator (180 rpm) at 28 °C for 48 h. Bacterial cells were harvested by centrifugation (3000 × g, 10 min), washed twice, and re-suspended with sterilized distilled water to a final concentration of 1 × 10^9^ CFU (colony forming unit) ml^−1^.

Four TC-1's infectious doses were prepared for white grub bioassays by mixing the bacterial suspension with sterilized soil (i.e. 1 × 10^9^, 5 × 10^8^, 2.5 × 10^8^, and 1.25 × 10^8^ CFU g^−1^). The amount of soil was based on the numbers of white grubs tested (Xu et al. 2009). The white grubs were placed individually in feeding boxes containing potato slices and the soil with the different TC-1's doses. The mortality rate was scored every three days. A larva was considered dead if no movement was detected after being stimulated with a blunt-ended tip. Each treatment consisted of 20 white grubs with three independent replicates. The soil treated with sterilized water alone was used as control (CK). A corrected mortality rate (RMR) was calculated as RMR = (MRT-MRCK)/(1-MRT) × 100, where MRT and MRCK represent the mortality rate of treatment and the mortality rate of CK, respectively.

In the bioassays with *B. mori*, fresh mulberry leaves were washed with tap water, air-dried, sprayed with 1 × 10^9^ CFU ml^−1^ of a TC-1's suspension, and used for feeding larvae from second- and third-instar stages. Mortality rates were scored daily. Leaves sprayed with water alone were used as CK. Each treatment consisted of 20 larvae with four independent replicates.

The virulence of strain TC-1 to *H. armigera*, *P. xylostella*, *S. exigua* and *S. litura* was tested by feeding second- and third-instar larvae with artificial diet portions (approximately 0.2 × 0.2 × 0.2 cm^3^) that were previously dipped in a TC-1's suspension (1 × 10^9^ CFU ml^−1^). Mortality rates were scored daily. Diets dipped in sterilized water alone were used as CK. Each treatment consisted of 20 larvae with four independent replicates. The larvae of *H. armigera*, *S. exigua* and *S. litura* were reared individually to prevent cannibalism.

The virulence of strain TC-1 on *C. elegans* was tested using the method previously described by Niu et al. (2010) with a slight modification. Autoclaved cellophane paper was used to cover the PBA medium (peptone, 10 g l^−1^; beef extract, 3 g l^−1^; dextrose, 10 g l^−1^; NaCl, 5 g l^−1^; agar, 16 g l^−1^). A TC-1's suspension (10^6^ CFU ml^−1^) was spread on the cellophane paper and the plates incubated at 28 °C for 3 d. One drop (50 μl) of a *C. elegans* suspension containing approximately 1000 worms from second- and third-instars was placed on the TC-1 lawn. The number of dead worms was counted under a stereomicroscope at the three incubation times (36, 48 and 72 h). The nematodes were considered dead if no movement was detected after being stimulated gently with a stick. The experiment was independently replicated three times.

### Preparation of colloidal chitin

Colloidal chitin was prepared by adding concentrated hydrochloric acid (36–38%, 100 ml) to 15 g of powdered chitin (Beijing Solarbio Science & Technology Co., Ltd.) followed by continuous stirring at 4 °C. After stirring for 20 min, the chitin was precipitated as a colloidal suspension by slowly adding 2 l of distilled water at 4 °C. The precipitate was collected and treated again with concentrated hydrochloric acid as described above. The resultant precipitate was re-suspended in 100 ml of distilled water, mixing carefully to produce a solution with butyrous consistency containing 1.5% (m/v) colloidal chitin. The pH of the colloidal chitin was adjusted to 7 using a sodium hydroxide solution.

### Chitinase activity assay

The chitinase activity was tested by detecting N-acetylglucosamine (NAG) as the final product using a routine method (Moon et al. 2017; Abudunasier et al. 2019) with a slight modification. Briefly, 1-ml of the bacterial suspension was centrifuged (10,000 × g, 10 min) and 0.4 ml of the culture supernatant mixed with an equal amount of 1.5% (m/v) colloidal chitin solution followed by incubation at 30 °C in a water bath for 30 min. Then, 0.6 ml of distilled water and 3,5-dinitrosalicylic acid (DNS) reagent were added separately to stop the reaction, followed by heating at 100 ºC for 5 min. After centrifugation (10,000 × g, 10 min), reducing sugar in the supernatant was determined by measuring the absorbance at 540 nm using an UV spectrophotometer (PRESEE ANALYTICS, TU-1901). The DNS reagent was prepared by dissolving 36.4 g of Seignette salt in 100 ml of distilled water and heating until it was completely dissolved. Then, DNS (1.26 g), sodium hydroxide (4.20 g), and crystal phenol (1 g) were dissolved separately into the Seignette salt solution, and the volume was made up to 200 ml with distilled water. A standard curve of NAG was constructed using the method described by Hu et al. (2016) with the regression equation Y = 0.1366X-0.1724, where “Y” and “X” represent OD_540_ and NAG concentration (mg l^−1^), respectively (Additional file 1: Fig. S1). The correlation coefficient (r) of the equation was 0.9971, showing a highly positive relationship between the NAG concentration in the solution and the OD_540_ value. One unit of chitinase activity per milliliter (U ml^−1^) was defined as the amount of the enzyme required for producing 1 µmol of NAG from chitin.

### Optimization of culture conditions for chitinase production

The effects of four carbon sources (colloidal chitin, powdered chitin, starch, and cellulose) on chitinase production by the strain TC-1 were tested. Ten gram of each carbon source were added separately to 1000 ml of PBB liquid medium (the same components as PBA, except agar) without dextrose. Strain TC-1 was inoculated into each medium containing different carbon sources and allowed to grow in a rotary incubator (180 rpm) at 28 °C for 60 h. The chitinase activities in the culture supernatants were separately determined using the method as described above. Three replicates were set up for each treatment.

The effects of four nitrogen sources (peptone, ammonium chloride, ammonium sulfate, and beef extract) on chitinase production by the strain TC-1 were tested. For the ammonium chloride and ammonium sulfate tests, both beef extract and peptone in the PBB liquid medium were replaced by a test nitrogen source. For the peptone test, the PBB liquid medium free of beef extract was used. For the beef extract test, the PBB liquid medium free of peptone was used. In all cases, the concentration of the nitrogen source was 10 g l^−1^. The strain TC-1 was inoculated into a test medium with a single nitrogen source and allowed to grow in a rotary incubator (180 rpm) at 28 °C for 60 h. The chitinase activities in the culture supernatants were determined separately using the method described above. Three replicates were set up for each treatment.

### Colloidal chitin concentration

Strain TC-1 was inoculated into the PBB liquid medium in which dextrose was substituted with colloidal chitin at different concentrations (7, 8, 9, 10, 11, 12, and 13 g l^−1^) and allowed to grow in a rotary incubator (180 rpm) at 28 °C for 60 h. The chitinase activities in the culture supernatants were determined separately using the method described above. Three replicates were set up for each treatment.

### Incubation time

Strain TC-1 was inoculated in PBB liquid medium and allowed to grow in a rotary incubator (180 rpm) at 28 °C for 24, 30, 36, 54, 60, 66, and 72 h, respectively. The chitinase activities in the culture supernatants were determined separately using the method described above. Three replicates were set up for each treatment.

### Incubation temperature

Strain TC-1 was inoculated in PBB liquid medium and allowed to grow in a rotary incubator (180 rpm) at different temperatures (24, 26, 28, 30, and 32 °C) for 60 h. The chitinase activities in the culture supernatants were determined separately using the method described above. Three replicates were set up for each treatment.

### Response surface design

The chitinase production by strain TC-1 was optimized with response surface methodology (RSM) using a three-factor (colloidal chitin concentration, incubation time and incubation temperature), three-level Box-Behnken experimental design. The three factors were selected based on single-factor experiments. The three levels were 8.2 g l^−1^, 8.95 g l^−1^ and 9.7 g l^−1^ for the colloidal chitin concentration, and 58 h, 64 h and 70 h for the incubation time, and 26 °C, 28 °C and 30 °C for the incubation temperature, respectively (Additional file 1: Table S5).

## Results

### Morphological, physiological, and biochemical properties of strain TC-1

**Fig. 1:**
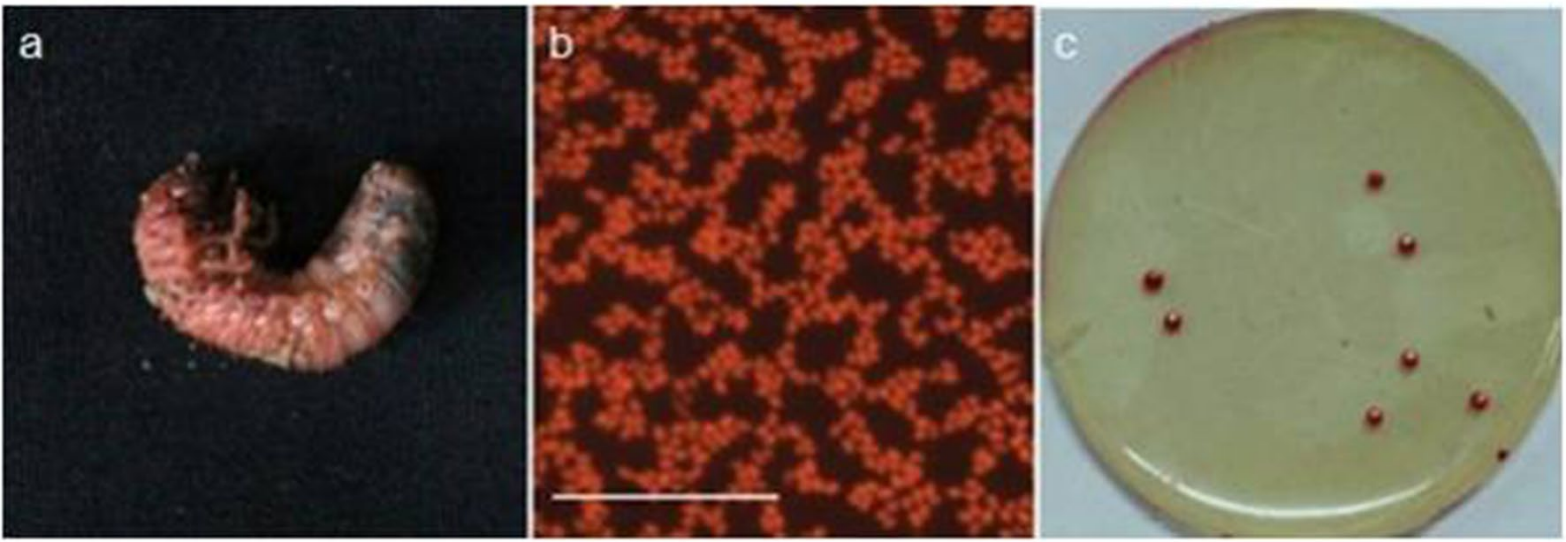
Symptoms of infection by *S. marcescens* TC-1 and morphological characteristics of strain TC-1. **a** Symptoms of TC-1 infection in white grub; **b** TC-1 bacterial cells (scale bar = 10 μm); **c** TC-1 rose red colonies grown on a BPYDA plate (7 mm in diameter) after 48 h at 28 ℃

As shown in Fig. 1b and c, TC-1 was a Gram-negative, short rod-shaped, non-sporulating bacterium with peritrichous flagella and fluorochrome. When grown on BPYDA media at 28 °C for 48 h, the resultant colonies were round, rose red, 2–3 mm in diameter, with a moist glassy surface.

The results of the physiological and biochemical characterization of TC-1 are summarized in Table 1. Of the 28 physiological and biochemical items tested, positive reactions were observed in the 16 items (motility, utilization of mannitol, sorbitol, adonitol, xylose and sucrose, acid production from glucose, DNase, lysine decarboxylase, ornithine decarboxylase, arginine decarboxylase, bile esculin, V.-P., gelatin liquefaction, Simmons citrate, reduction of NO_3_^−^ to NO_2_). The remaining 12 items (oxidase, urease, phenylalanine deaminase, methyl red, production of H_2_S and indole, utilization of melibiose, lactose, raffinose and L-arabinose and malonate) were negative.

**Table 1:** Physiological and biochemical characteristics of strain TC-1

Trait	Response	Trait	Response
Oxidase	−	Phenylalanine deaminase	−
Gram staining	−	Lysine decarboxylase	+
Motility	+	Ornithine decarboxylase	+
H_2_S production	−	Arginine decarboxylase	+
Mannitol	+	Bile esculin	+
Urease	−	Methyl red	−
Indole production	−	Sucrose	+
Melibiose	−	Acid from glucose	+
Sorbitol	+	Voges-Proskauer	+
DNase	+	Raffinose	−
Lactose	−	L-Arabinose	−
Adonitol	+	Gelatin liquefaction	+
Xylose	+	Simmons citrate	+
NO3^−^ → NO2^−^	+	Malonate	−

Note: “ + ” and “−” represent positive and negative reactions, respectively

### Molecular identification and phylogenetic analysis of strain TC-1

**Fig. 2:**
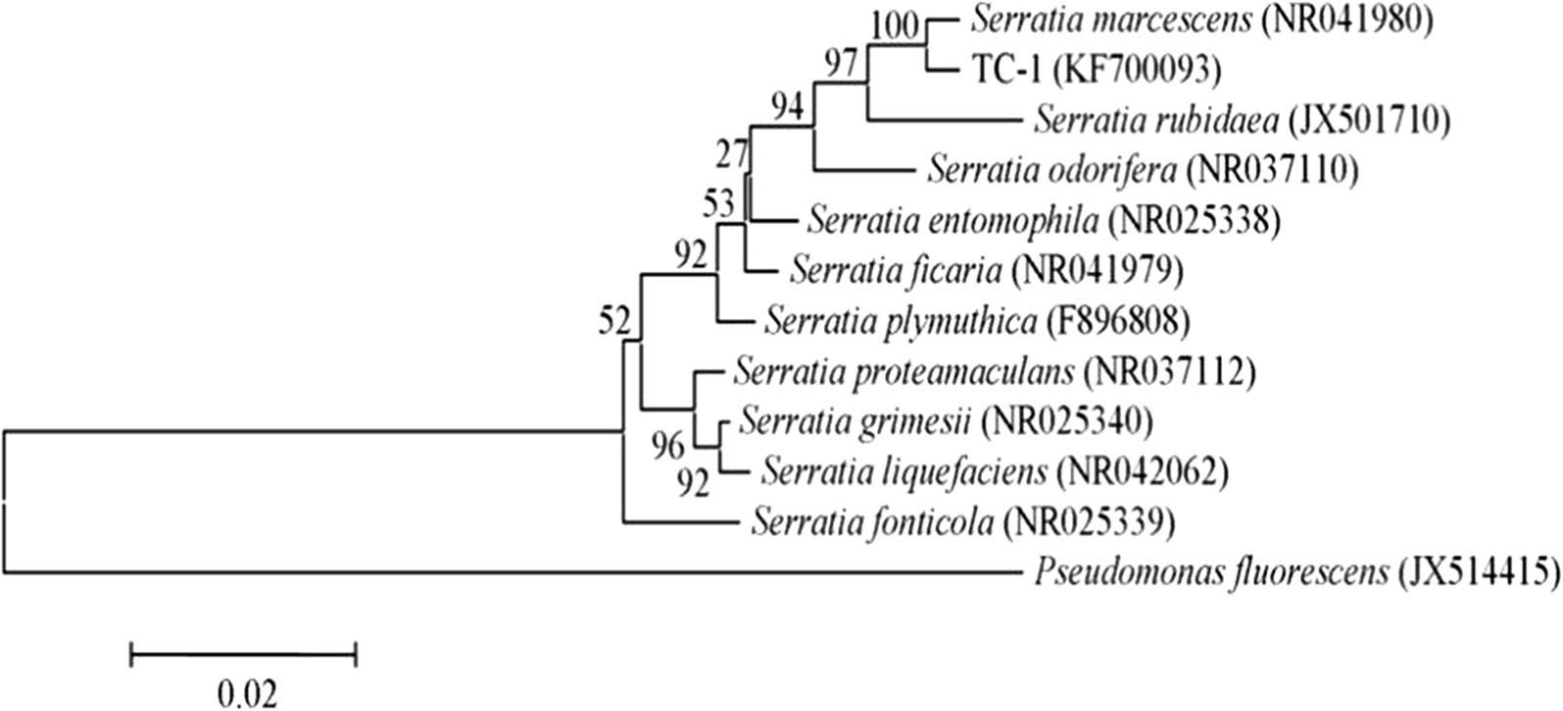
The MEGA7.0-constructed Neighbor-joining phylogenetic tree based on 16S rRNA sequences showing the genetic relationship between strain TC-1 and other *Serratia* spp. retrieved from the GenBank database. The numbers in parentheses represent the accession numbers in GenBank. The values (from 1000 replicates) are indicated at the branch nodes as the percentages supported by bootstrap. *Pseudomonas fluorescens* (JX514415) was used to root the phylogenetic tree. The scale bar represents a genetic distance of 0.02 substitutions per nucleotide position

The amplified sequence of TC-1's 16S rRNA gene was 1448 base pairs in length and was deposited in GenBank (accession number KF700093). BLAST analysis showed a sequence identity greater than 99% to multiple strains of *S. marcescens*. In the MEGA 7.0-reconstructed Neighbor-joining phylogenetic tree of bacterial 16S rRNA gene sequences, strain TC-1 clustered with *S. marcescens* (GenBank acc. no. NR041980) at 100% bootstrap level, clearly separated from other *Serratia* spp. (Fig. 2). The molecular data conclusively supported strain TC-1 as a member of *S. marcescens*.

### Virulence of strain TC-1 against insects and nematode

The white grubs (*A. corpulenta*) began to die 48 h after exposure to the bacterium TC-1, with mortality peaking at 84 h. The infected insects turned inactive and stopped feeding. The white grub cadavers turned reddish-brown and gradually softened, losing all their elasticity (Fig. 1a). Bacterial cultures with similar phenotypic features to the inoculum could be isolated from surface-disinfected dead grubs, confirming strain TC-1 as a pathogen of *A. corpulenta* based on Koch’s postulates. The bacterial concentration significantly influenced the mortality rate of white grubs in the soil. The highest mortality rate (91.7%) was observed at the higher concentration of the inoculum (1 × 10^9^ CFU ml^−1^) (Table 2). A regression equation modeling the relationship between grub mortality rate (Y) and TC-1 concentration (X) was obtained as Υ = 5.5287 + 1.3010Χ, with correlation coefficient (r) equal to 0.9619.

**Table 2:** Virulence of strain TC-1 to white grubs (*Anomala corpulenta*)

Concentration of strain TC-1 (108 CFU ml^−1^)	Number of white grub tested	Number of dead white grub			Mortality (%)	Corrected mortality (%)	LogC(Y)	Fatal probability(X)
		Test1	Test2	Test3				
10 ×	20	18	19	18	91.7 a	91.1 a	9	6.3469
5 ×	20	16	16	15	78.3 b	76.7 b	8.699	5.7290
2.5 ×	20	12	11	14	61.7 c	58.9 c	8.3979	5.2250
1.25 ×	20	10	9	10	48.3 d	44.6 d	8.0969	4.8642
0.625 ×	20	8	10	10	46.7 d	42.9 d	7.7959	4.8211
CK	20	1	1	2	6.7 e			

Data with different lowercases in each column indicate significant difference at *P* < 0.05 level

More than 95% of *B. mori* larvae fed with TC-1-sprayed mulberry leaves showed anorectic behavior, and became inactive and insensitive to external stimulation within the first 24 h. During the next 24 h, most larvae stopped feeding completely. After 48 h, successive deaths were recorded in treated larvae. *B. mori* larval mortality rate reached 88.6% and 100% after 96 and 144 h of exposure to TC-1-sprayed mulberry leaves, respectively (Table 3). However, no bacterial culture with TC-1's phenotypic features could be re-isolated from the surface-disinfected *B. mori* cadavers.

**Table 3:** Virulence of strain TC-1 to insect larvae and *Caenorhabditis elegans*

Target organism	Treatment	Mortality (%)						96 h Corrected mortality (%)
		24 h	48 h	72 h	96 h	120 h	144 h	
*C. elegans*	Test	20.0	72.2	93.3	100	−	−	100
	CK	1	2.0	4.0	4	4	4	
*B. mori*	Test	0	18.3	41.2	86.6	98.3	100	86.4
	CK	0	1.6	1.6	1.6	1.6	5.0	
*P. xylostella*	Test	20.0	48.9	71.2 ( +)	75.3 ( +)	+	+	72.2
	CK	1.7	6.3	6.3	11.3	+	+	
*S. exigua*	Test	15.0	92.5	95.0	95.0	95.0	95.0	94.6
	CK	5.0	7.5	7.5	7.5	7.5	7.5	
*H. armigera*	Test	3.8	26.3	60.0	85.0	90.0	92.5	83.8
	CK	0	0	2.5	7.5	11.3	13.5	
*S. litura*	Test	0	0	0	0	0	0	
	CK	0	0	0	0	0	0	0

Note: “ + ” and “−” represent “pupation” and “termination of observation”, respectively

*H. armigera*, *P. xylostella*, *S. exigua*, and *S. litura* larval mortality rates were investigated between 24 and 144 h of TC-1 exposure. During this time, successive mortality was recorded in *H. armigera*, *P. xylostella* and *S. exigua* larvae, with corrected mortality rates of 83.8%, 72.2% and 94.6%, respectively (Table 3). The peak mortality rates for *P. xylostella* and *H. armigera* occurred within 76 h and 144 h of TC-1 exposure, respectively. *P. xylostella* larvae that survived bioassays started premature pupation after 76 h of TC-1 exposure. No death due to TC-1 was recorded in *S. litura* larvae within 144 h of TC-1 exposure.

*C. elegans* was highly susceptible to the strain TC-1 and its larval mortality rate reached 100% after 96 h of exposure to the bacterium (Table 3). Some worm cadavers turned reddish-brown, while others displayed no significant change in body color.

### Effects of culture conditions on chitinase production by strain TC-1

A total of four variables (carbon source, nitrogen source, incubation time, and incubation temperature) were analyzed regarding their effects on chitinase production by the strain TC-1 (Table 4). The results revealed peak chitinase activities of 18.89 U ml^−1^ when colloidal chitin was used as the sole carbon source, 17.8 U ml^−1^ when peptone was used as the sole nitrogen source, 17.44 U ml^−1^ at 60 h of incubation time, and 17.33 U ml^−1^ at the incubation temperature of 28 °C, respectively. Regarding the colloidal chitin concentration in the medium, the highest chitinase activity (20.79 U ml^−1^) was recorded with 9.5 g l^−1^ of colloidal chitin compared to the other concentrations tested (7, 8, 8.5, 9, 10, and 10.5 gl^−1^).

**Table 4:** Effects of culture conditions on chitinase production by strain TC-1

Items tested	Chitinase activity (U ml^−1^)
Carbon source	
Coloidal chitin	18.89 ± 0.04 Aa
Chitin	12.69 ± 0.04 Bb
Starch	3.09 ± 0.06 Cd
Celullose	3.28 ± 0.03 Cc
Nitrogen source	
Peptone	17.8 ± 0.17 Aa
Ammonium sulphate	9.49 ± 0.14 Cc
Yeast extract	15.37 ± 0.25 Bb
Beef extract	15.16 ± 0.19 Bb
Incubation time (h)	
24	7.75 ± 0.24 Gh
30	8.2 ± 0.11 Fg
36	8.45 ± 0.11 Fg
42	9.98 ± 0.23 Ef
48	10.44 ± 0.05 De
54	12.81 ± 0.13 Cc
60	17.44 ± 0.07 Aa
66	14.69 ± 0.28 Bb
72	12.47 ± 0.19 Cd
Incubation temperature (°C)	
24	10.55 ± 0.06 Cc
26	14.91 ± 0.09 Bb
28	17.33 ± 0.17 Aa
30	14.92 ± 0.15 Bb
32	9.69 ± 0.08 Cd
Colloidal concentration (g l^-1^)	
7.5	13.08 ± 0.25 Ee
8.0	14.39 ± 0.16 Dd
8.5	18.51 ± 0.09 Bb
9.0	18.68 ± 0.08 Bb
9.5	20.79 ± 0.19 Aa
10.0	16.43 ± 0.10 Cc
10.5	10.91 ± 0.10 Ff

The analysis of variance for each item was independently tested. Data with different capitals and lowercases in the same item represent significant differences at *P* < 0.01 and *P* < 0.05 levels, respectively

### RSM optimization of TC-1 culture conditions for chitinase production

Based on the results of single-factor experiments, the three most significant factors for chitinase production by TC-1, i.e. colloidal chitin concentration, incubation time and incubation temperature, were selected for further optimization using a three-factor, three-level RSM design (Table 5). The response of chitinase production (*Y*) by the strain TC-1 could be expressed by the following quadratic regression model:

**Table 5:** The chitinase activities in the culture supernatants of strain TC-1 under different combinations of three factors at different levels

Serial number	A (g l^−1^)	B (h)	C (°C)	Y (U ml^−1^)
1	9.70	64.00	26.00	16.43
2	8.95	64.00	28.00	20.72
3	8.95	58.00	30.00	16.13
4	9.70	70.00	28.00	15.70
5	8.95	64.00	28.00	20.88
6	8.95	70.00	30.00	15.97
7	8.20	58.00	28.00	14.87
8	8.95	58.00	26.00	15.21
9	9.70	64.00	30.00	17.06
10	8.20	64.00	30.00	15.28
11	9.70	58.00	28.00	16.79
12	8.95	64.00	28.00	21.06
13	8.95	70.00	26.00	15.59
14	8.95	64.00	28.00	20.97
15	8.95	64.00	28.00	20.88
16	8.20	70.00	28.00	14.80
17	8.20	64.00	26.00	15.21

The three factors “A”, “B” and “C” represent chitin concentration, incubation time and incubation temperature, respectively. “Y” represents chitinase activity

Y = 20.90 + 0.73A-0.12B + 0.25C − 0.26AB + 0.14AC − 0.13BC − 2.55A^2^ − 2.82B^2^ − 2.36C^2^, where A is the colloidal chitin concentration, B is the incubation time; and C is the incubation temperature.

The analysis of variance (ANOVA) of the quadratic regression model was performed (Table 6). The *P*values for the model (< 0.0001) and "lack-of-fit" (0.0579), suggested that the established regression model was appropriate without significant deviation. The greater the *F* value was from unity and the lower the *P*-value was, the greater the effect of the tested factor on chitinase production by TC-1. The *P*-value of A (< 0.0001) indicated the effect of colloidal chitin concentration on chitinase production by the bacterium was highly significant. The *P*-value of C (0.016) indicated that the influence of temperature on chitinase production by the bacterium was significant. Consequently, the order of influence of the three factors on chitinase production, from the largest to the smallest, was colloidal chitin concentration (A), incubation temperature (C) and incubation time (B) in sequence. The *P-*values of the three factors' combinations AB, AC and BC were 0.0568, 0.2512 and 0.2669, respectively, indicating that the difference of reciprocal effects of the three factors A, B and C on chitinase production by TC-1 was not significant. The *P-*values of A^2^, B^2^ and C^2^ were all less than 0.0001, indicating that the quadratic terms of the three factors A, B and C had a significant influence on chitinase production by the bacterium.

**Table 6:** Analysis of variance for the regression model of chitinase activity

Source of variation	Degree of freedom	F-value	*P*-value	Significance of difference
Model	9	220.03	< 0.0001	**
A	1	84.49	< 0.0001	**
B	1	2.20	0.1812	
C	1	9.98	0.0160	*
AB	1	5.19	0.0568	
AC	1	1.56	0.2512	
BC	1	1.45	0.2669	
A^2^	1	544.65	< 0.0001	**
B^2^	1	666.29	< 0.0001	**
C^2^	1	468.37	< 0.0001	**
Residual	7			
Lack-of-fit	3	6.01	0.0579	
Pure error	4			
Cor total	16			

**Fig. 3:**
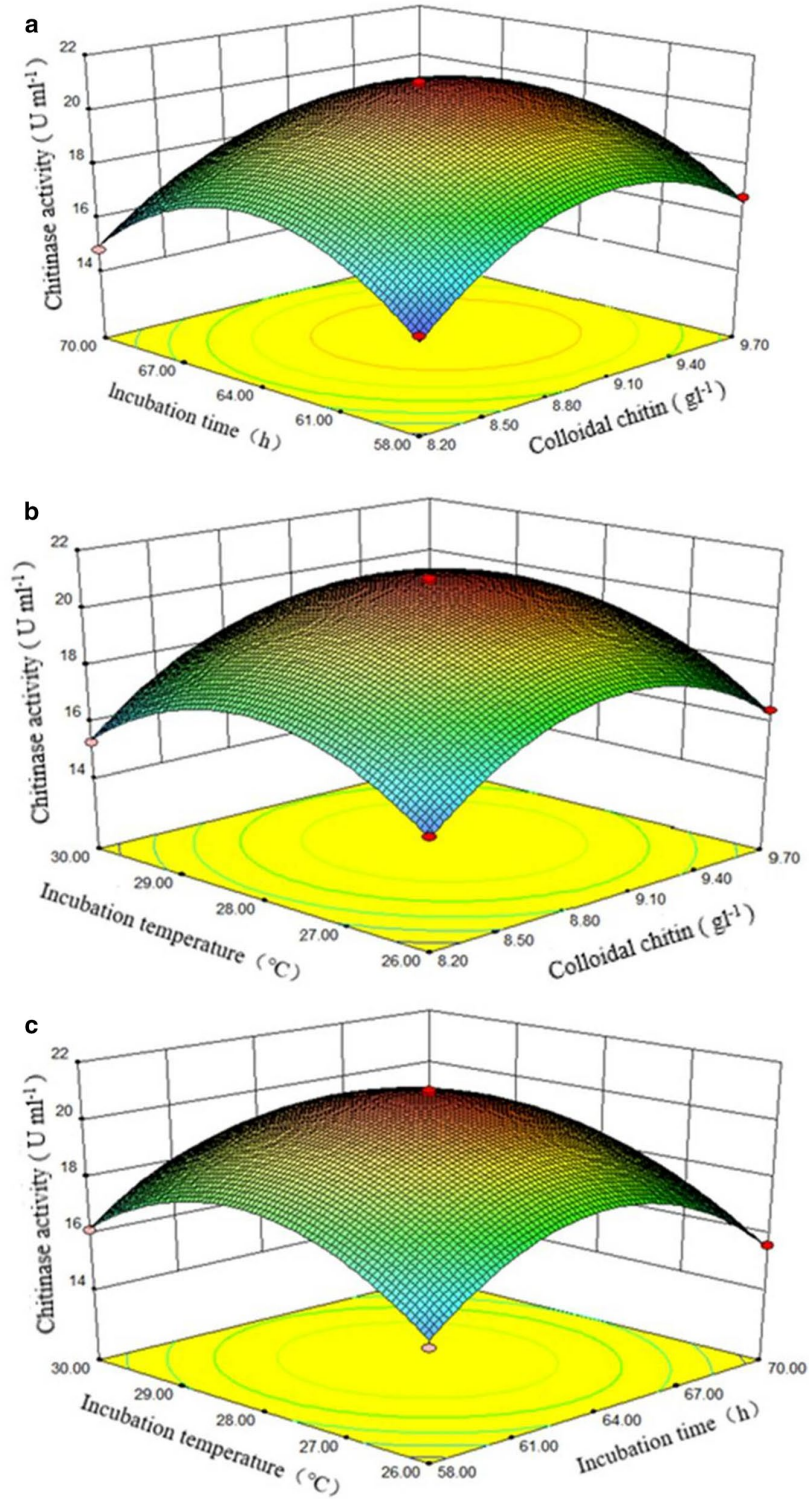
Response surface and counter plots of the interactive effects on chitinase production by the strain TC-1 between colloidal chitin concentration and incubation time (**a**), colloidal chitin concentration and incubation temperature (**b**), and incubation time and incubation temperature (**c**)

The credibility analysis of the regression model was performed (Additional file 1: Table S6). The standard deviation and the coefficient of variation of the regression model were 0.22 and 1.3, with PRESS (prediction sum of squares) and “signal-to-noise” ratio being 4.69 and 36.269, respectively. The multiple correlation coefficient, the predicted correlation coefficient and the corrected correlation coefficient were 0.9965, 0.9529 and 0.9919, respectively, indicating high fit of the constructed regression model. Based on the regression analysis and fitness of the constructed regression model, three RSM diagrams were obtained (Fig. 3). The chitinase activity peaked at the appropriate combination of colloidal chitin concentration and incubation time (Fig. 3a), colloidal chitin concentration and incubation temperature (Fig. 3b), or incubation time and incubation temperature (Fig. 3c). Based on the analyses using software Design-Expert 8.0 (Stat-Ease Inc., Minneapolis, MN, USA), the predicted optimal chitinase activity was 20.946 U ml^−1^ at a combination of colloidal chitin concentration, incubation time, and incubation temperature of 9.06 g l^−1^, 63.83 h, and 28.12 °C, respectively. The mean chitinase activity measured under laboratory conditions was 20.761 ± 0.102 U ml^−1^ at a combination of colloidal chitin concentration, incubation time, and incubation temperature of 9 g l^−1^, 64 h and 28 °C, respectively, for three replicates. No significant differences were detected between the predicted optimal chitinase activity and the actual activity measured in the laboratory, indicating that the proposed regression model is reliable.

## Discussion

Biological control of insect pests represents an important eco-friendly agronomic measure for sustainable agriculture and forestry. Successful biocontrol of an insect pest depends largely on the acquisition of microbial strains with high insecticidal activity. Many *S. marcescens* strains can colonize the alimentary canal of insects and induce their death by septicemia. Infections of insects by *S. marcescens* have been reported in numerous pest species that attack aboveground parts of crops, such as *H. armigera* (Chen et al. 2005; Shi et al. 2003), *Oedaleus infernalis* (Feng et al. 2002; Jin et al. 2005), *Diatraea saccharalis* (King et al. 1975), *Heliothis virescens* (Sikorowski et al. 2001), *Lymantria dispar* (Podgwaite and Cosenza 1976), *Anoplophora glabripennis* (Deng et al. 2008), *Phyllotreta striolata* (Yang et al. 2014a, b), *S. exigua* (Qi et al. 2004; Yang et al. 2015a, b; Zhao et al. 2017), *S. litura* (Niu et al. 2015), *Heortia vitessoides* (Tan and Zhang 2005), *Rhynchophorus ferrugineus* (Zhang et al. 2011) and *Orthaga achatina* (Guan et al. 2018). In the present study, strain TC-1 was isolated from a naturally infected larva (white grub) of the underground pest species (*A. corpulenta*).

Both the physiochemical and molecular data conclusively supported strain TC-1 as a member of *S. marcescens*. The bacterial isolates with the ability to ferment glucose, liquefy gelatin rapidly, oxidase and phenylalanine-deaminase negative and cannot acidify arabinose and raffinose in peptone water under aerobic conditions are generally accepted as *S. marcescens* (Anderhub et al. 1977). The physiochemical reactions of strain TC-1 agreed well with these *S. marcescens-*specific features. In the Neighbor-joining phylogenetic tree of bacterial 16S rRNA gene sequences, the strain TC-1 formed a clade with *S. marcescens*, clearly separated from other *Serratia* spp.

Previously, Niu et al. (2015) reported an *S. marcescens* strain S-JS1 virulent against two lepidopteran species *S. exigua* and *S*. *litura*. In our study, pathogenicity tests revealed the broad insecticidal spectrum of *S. marcescens* TC-1, including an underground Coleoptera pest *A. corpulenta* and four species of Lepidoptera (*B. mori*, *P. xylostella*, *S. exigua*, and *H. armigera*). Interestingly, TC-1 showed no toxicity to another *Spodoptera* species (*S. litura*), suggesting the existence of host species-specific interactions between the virulence factors in TC-1 and the insects’ innate immune system. Further comparison between the compatible *S. exigua*-TC-1 and incompatible *S. litura*-TC-1 interactions at the molecular level could provide valuable insights into the mechanism(s) of virulence of the strain TC-1. Little is known about *S. marcescens* strains with the potential to control underground insect pests. This study is the first on a *S. marcescens* strain with the potential to control the larvae of *A. corpulenta*, which damage the root of many crops. The larvicidal activity of *S. marcescens* TC-1 against *C. elegans* also shows the potential of this bacterium as a biocontrol agent against plant diseases caused by nematodes.

Chitinase is an important enzyme for the control of insect pests as well as fungal plant pathogens (Oppenheim and Chet 1992). Previous studies have shown that *S. marcescens* produced multiple chitinase isozymes. Horn et al. (2006) reported that *S*. *marcescens* could produce several chitinolytic enzymes, including chitinases A, B and C, which enable the bacterium to degrade the insoluble chitin polymer efficiently. Someya et al. (2001) discovered four chitinolytic enzymes among the extracellular proteins produced by *S. marcescens* strain B2, and found both chitinolytic enzymes and prodigiosin could act synergistically against the grey mold pathogen, *Botrytis cinerea*. Watanabe et al. (1997) reported four chitinases, A, B, C1, and C2 in the culture supernatant of *S. marcescens* 2170. Fuchs et al. (1986) found that *S. marcescens* produced five unique chitinolytic proteins with subunit molecular masses of 21, 36, 48, 52, and 57 kDa. Jones et al. (1986) characterized the genes encoding two chitinase enzymes of *S. marcescens*. Having established that the isoelectric point of the primary chitinase produced by the strain TC-1 was pH 6.7 (Additional file 1: Tables S2 and S3), we applied selective isoelectric point precipitation to CS of TC-1. In initial tests, we then confirmed that this chitinase-enriched CS showed larvicidal activity against *S. exigua* (Additional file 1: Table S4). Further purification and enzymatic characterization of the chitinase(s) produced by the strain TC-1 is required for a full understanding of its virulence against the insect species tested and *C. elegans*. Although the number of chitinases produced by strain TC-1 remains to be specified, we speculate that the lack of insecticidal activity of *S. marcescens* TC-1 against *S. litura* may be due to the fact that the chitinase isoforms in this strain differ from those of the *S. litura*-toxic *S. marcescens* S-JS1 isolate reported by Niu et al. (2015).

In our study, an intriguing issue was the nature of the bacterial factor(s) that caused a high prevalence (95%) of TC-1-induced anorexia in *B. mori* larvae during the first stage of the insect death. However, the impossibility of re-isolating the strain TC-1 from exposed insects' cadavers suggests no multiplication of TC-1 within the insect body. We speculated that strain TC-1 could directly produce volatiles that induced anorexia in *B. mori* larvae and/or produce substance(s) that trigger the release of volatiles from the mulberry leaves that induced anorexia in the insect larvae*.* The absence of TC-1 in *B. mori* cadavers could be reasonably explained as a consequence of the insect’s anorexia.

Huang et al. (2010) reported RSM-optimized culture conditions for chitinase production by *S. marcescens* strain S418 based on a combination of 0.2% colloidal chitin, 1% peptone and 0.05% KH_2_PO_4_. In our study, a concentration of 9 g l^−1^ of colloidal chitin was optimal for strain TC-1. This concentration is significantly higher than the reported by Huang et al. (2010) for strain S418 and could be associated with strain-specific differences in the efficiency of utilizing colloidal chitin as a carbon source.

We established a quadratic regression model with a multiple correlation coefficient and a corrected correlation coefficient of 0.9965 and 0.9919, respectively. The difference between the predicted chitinase activity (20.946 U ml^−1^) and that actually measured in the laboratory (20.761 ± 0.102 U ml^−1^) was only 0.201 U ml^−1^, revealing that our RSM-developed quadratic regression model has the potential to guide chitinase production by strain TC-1 under laboratory conditions. Further studies and revisions of the regression model may be required for large-scale chitinase production by TC-1.

## Supplementary Information

**Additional file 1: Table S1**. Effects of two levels (A and B) of chitinase-containing culture supernatants (CCS) of strain TC -1 on death rates of *Spodoptera exigua* larvae. **Table S2**. A broad-spectrum screening assay of primary chitinase with the highest chitinolytic activity in the culture supernatant of the strain TC-1 by isoelectric precipitation. **Table S3**. A narrow-spectrum screening assay of primary chitinase with the highest chitinolytic activity in the culture supernatant of the strain TC-1 by isoelectric precipitation. **Table S4**. Toxicity of the pH 6.7-precipitated crude chitinase from the culture supernatant of strain TC-1 against the second- and third-instar larvae of *Spodoptera exigua1*. **Table S5**. Factors and levels of response surface design for optimization of chitinase production conditions of strain TC-1. **Table S6**. Credibility analysis of the regression model. **Figure S1**. The standard curve of N-acetylglucosamine.

## Data Availability

The raw sequencing data of the 16S rRNA gene of strain TC-1 have been submitted to the NCBI, and the sequence read archive number (accession number) was KF700093. The other datasets generated during and/or analyzed during the study are available from the corresponding author on reasonable request.
