# Supplementary material for: Characterization of a novel chitinolytic Serratia marcescens strain TC-1 with broad insecticidal spectrum
Source: AMB Express. 2022 Jul 30;12:100. doi: 10.1186/s13568-022-01442-6 (PMC9339060; doi:10.1186/s13568-022-01442-6)
Supplement: Supplementary file 1 — Additional file 1: Table S1. Effects of two levels (A and B) of chitinase-containing culture supernatants (CCS) of strain TC -1 on death rates of Spodoptera exigua larvae. Table S2. A broad-spectrum screening assay of primary chitinase with the highest chitinolytic activity in the culture supernatant of the strain TC-1 by isoelectric precipitation. Table S3. A narrow-spectrum screening assay of primary chitinase with the highest chitinolytic activity in the culture supernatant of the strain TC-1 by isoelectric precipitation. Table S4. Toxicity of the pH 6.7-precipitated crude chitinase from the culture supernatant of strain TC-1 against the second- and third-instar larvae of Spodoptera exigua1. Table S5. Factors and levels of response surface design for optimization of chitinase production conditions of strain TC-1. Table S6. Credibility analysis of the regression model. Figure S1. The standard curve of N-acetylglucosamine. [file 13568_2022_1442_MOESM1_ESM.pdf]

# The Supplementary Materials

**Journal Name:**

AMB Express

**Manuscript Title:**

Characterization of a novel chitinolytic *Serratia marcescens* strain TC-1 with broad insecticidal spectrum

**The names of the authors:**

Aili Tao · Tan Wang · Fahu Pang · Xueling Zheng · Camilo Ayra-Pardo · Siliang Huang · Ruxin Xu · Fengqin Liu · Jiakang Li · Yibin Wei · Zhiqing Wang · Qiuhong Niu · Dandan Li

**The affiliation and address of the authors:**

School of Life Science and Agricultural Engineering, Nanyang Normal University, Nanyang 473061, China

**The e-mail address and telephone number of the corresponding author:**

Siliang Huang

silianghuang@aliyun.com

86(0)15839924714

**Table S1.** Effects of two levels (A and B) of chitinase-containing culture supernatants (CCS) of strain TC -1 on death rates of *Spodoptera exigua* larvae.

| **Treatments**[1] | **Cumulative death rate (%) over CCS exposure time (h)** | | | | | | |
|---|---|---|---|---|---|---|---|
| | **24** | **48** | **72** | **96** | **120** | **144** | **178** |
| CCS-A | 6.7 | 16.7 | 20.0 | 20.0 | 23.3 | 63.3 | 100 |
| CCS-B | 3.3 | 16.7 | 40.0 | 46.0 | 50.0 | 86.7 | 100 |
| Cell-free culture broth (Control) | 0.0 | 6.7 | N/A | 10.0 | 10.0 | 20.0 | 40.0 |

[1] Level A (CCS-A) corresponds to a dilution ratio of CCS : Cell-free culture broth of 0.25 : 0.75, while level B (CCS-B) corresponds to a dilution ratio of CCS : Cell-free culture broth of 1:1. A cell-free culture broth was used as a control.

**Table S2** A broad-spectrum screening assay of primary chitinase with the highest chitinolytic activity in the culture supernatant of the strain TC-1 by isoelectric precipitation[1].

| pH | Chitinolytic activity (U $ml^{-1}$)[2] |
|---|---|
| 4.5 | 0.85 |
| 4.8 | 2.90 |
| 5.1 | 2.78 |
| 5.4 | -3.24 |
| 5.7 | 2.90 |
| 6.0 | -16.08 |
| 6.3 | 1.53 |
| 6.6 | 12.22 |
| 6.9 | 3.01 |
| 7.2 | -7.22 |
| 7.5 | -3.01 |
| 7.8 | 6.31 |
| 8.1 | -1.08 |
| 8.4 | 2.67 |
| 8.7 | -0.63 |
| 9.0 | 1.76 |

[1]To produce a chitinase-containing culture supernatant, TC -1 was inoculated in a chitin nutrition broth (CNB) consisting of 3 g beef extract, 10 g peptone, 9 g colloidal chitin, 10 g sucrose and 5 g NaCl in 1000 ml distilled water (pH 7.0) and incubated for 64 h at 28°C in a rotary incubator (150 rpm). The resultant bacterial suspension was centrifuged (3000 ×g, 10 min) to obtain a chitin-containing culture supernatant. Ten ml of supernatant was mixed with an equal volume of a phosphate buffer solution (PBS) at a specified pH. The final pH of the supernatant was adjusted to the target pH using a pH meter. After 30 min of static precipitation, the resultant precipitate was collected by centrifugation (3000 ×g, 10 min) and dissolved in 5 ml of PBS (0.2M, pH 8.0) before measuring the chitinolytic activities of the precipitated protein.

[2] The chitinolytic activity of the unknown protein(s) in PBS (0.2M, pH 8.0) was determined using the regression equation Y=0.011X-0.0255, where "Y" and "X" represent OD540 and NAG (N-acetylglucosamine) concentration (mg $l^{-1}$). The correlation coefficient (r) of the equation was 0.9974. One unit of chitinase activity per milliliter (U $ml^{-1}$) was defined as the amount of enzyme required to produce 1 μmol NAG from chitin.

**Table S3.** A narrow-spectrum screening assay of primary chitinase with the highest chitinolytic activity in the culture supernatant of the strain TC-1 by isoelectric precipitation[1].

| pH | Chitinolytic activity (U $ml^{-1}$)[2] |
|---|---|
| 6.3 | 2.22 |
| 6.5 | 1.08 |
| 6.7 | 15.74 |
| 6.9 | 3.24 |
| 7.1 | -13.69 |
| 7.3 | -0.52 |

[1]To produce a chitinase-containing culture supernatant, TC-1 was inoculated in CNB and incubated for 64 h at 28°C in a rotary incubator (150 rpm). The resultant bacterial suspension was centrifuged (3000 ×g, 10 min) to obtain a chitin-containing culture supernatant. Ten ml of supernatant was mixed with an equal volume of a phosphate buffer solution (PBS) at a specified pH. The final pH of the supernatant was adjusted to the target pH using a pH meter. After 30 min of static precipitation, the resultant precipitate was collected by centrifugation (3000 ×g, 10 min) and dissolved in 5 ml of PBS (0.2M, pH 8.0) before measuring the chitinolytic activities of the precipitated protein.

[2] The chitinolytic activity of the unknown protein(s) in PBS (0.2M, pH 8.0) was determined using the regression equation Y=0.011X-0.0255, where "Y" and "X" represent OD540 and NAG (N-acetylglucosamine) concentration (mg $l^{-1}$). The correlation coefficient (r) of the equation was 0.9974. One unit of chitinase activity per milliliter (U $ml^{-1}$) was defined as the amount of enzyme required to produce 1 μmol NAG from chitin.

**Table S4.** Toxicity of the pH 6.7-precipitated crude chitinase from the culture supernatant of strain TC-1 against the second- and third-instar larvae of *Spodoptera exigua*[1]

| Treatment[2] | Number (percent) of dead larvae | | | Number (percent) of dead larvae in average[3] |
|---|---|---|---|---|
| | I | II | III | |
| A | 29 (96.7) | 28 (93.3) | 28 (93.3) | 28.3 (94.3) a |
| B | 29 (96.7) | 29 (96.7) | 29 (96.7) | 29.0 (96.7) a |
| C | 3 (10.0) | 6 (20.0) | 5 (16.7) | 4.7 (15.7) b |
| D | 9 (30.0) | 5 (16.7) | 7 (23.3) | 7.0 (23.3) b |

[1] To prepare a chitinase-containing culture supernatant, TC-1 was inoculated into CNB and incubated in a rotary incubator (150 rpm) at 28°C for 64 h. The bacterial supernatant was then added to the CNB. The resulting bacterial suspension was centrifuged (3000 ×g, 10 min) to obtain a chitin-containing culture supernatant. The chitinase in the collected culture supernatant was precipitated with a phosphate buffer solution (0.2M, pH 6.7). The resultant precipitate was collected by centrifugation (3000 ×g, 10 min) and dissolved in a phosphate buffer solution (0.2M, pH 8.0) that was half the amount of supernatant used. The protein with the highest chitinolytic activity precipitated at pH 6.7 was used as the crude chitinase for the experiment with three replicates ( Ⅰ， Ⅱ and Ⅲ). Thirty larvae of *S. exigua* were used for each replicate. The data in this table were recorded on the 4th day (96 h) of the four dietary treatments (A, B, C and D).

[2] Treatment A: The larvae of *S. exigua* were fed with an artificial diet supplemented with crude chitinase solution (0.2 ml $g^{-1}$). Treatment B: Larvae of *S. exigua* were fed an artificial diet supplemented with the original chitin-containing culture supernatant (0.2 ml $g^{-1}$) of the bacterium. The final chitinase activities in the diets of both treatments A and B were approximately 3.3 U $g^{-1}$. Treatment C: Larvae of *S. exigua* were fed an artificial diet supplemented with CNB (0.2 ml $g^{-1}$). Treatment D: The larvae of *S. exigua* were fed with the artificial diet alone.

[3] Means with the same letters are not significantly different according to Duncan's multiple range test at $P = 0.01$.

**Table S5.** Factors and levels of response surface design for optimization of chitinase production conditions of strain TC-1.

| Level | Factor | | |
|---|---|---|---|
| | A (Incubation time) /h | B (Incubation temperature) /°C | C (Colloidal chitin concentration) /g.l$^{-1}$ |
| -1 | 58 | 26 | 8.2 |
| 0 | 64 | 28 | 8.95 |
| 1 | 70 | 30 | 9.7 |

**Table S6.** Credibility analysis of the regression model.

| Item | Value | Item | Value |
|---|---|---|---|
| Standard deviation | 0.22 | Multiple correlation coefficient | 0.9965 |
| Mean | 17.27 | Corrected correlation coefficient | 0.9919 |
| Coefficient variation | 1.30 | Predicted correlation coefficient | 0.9529 |
| PRESS | 4.69 | Signal-to-noise ratio | 36.269 |

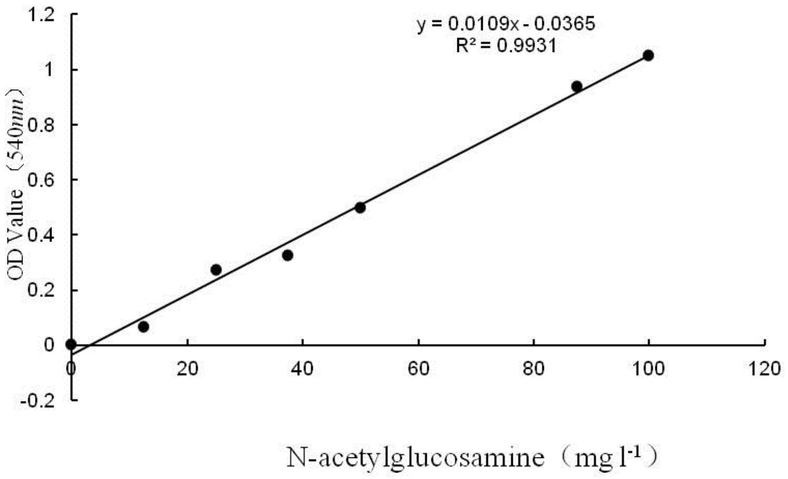


**Figure S1.** The standard curve of N-acetylglucosamine.

**The notification of acceptance of strain TC-1 as a culture collection in the China Center for Type Culture Collection.**

## China Center for Type Culture Collection

## 中 国 典 型 培 养 物 保 藏 中 心

### 用于专利程序的培养物保藏受理通知书（收据）

地址：中国. 武汉. 武汉大学　邮编：430072　电话：（027）68754052　传真：（027）68754833　E-mail:cctcc@whu.edu.cn

请求保藏人和其代理人：

请求保藏人：南阳师范学院　　陶爱丽

专利代理人：

专利申请号：

您（们）提供请求保藏的培养物名称及注明的鉴别特征：

粘质沙雷氏菌 TC-1
*Serratia marcescens* TC-1

本 保 藏 中 心 保 藏 编 号
CCTCC NO：M 2015634

上述请求保藏的培养物附有

☐ 科学描述
☑ 提议的分类命名

注：在框内打 √ 号表示有，打×号表示没有。

该培养物已于 2015 年 10 月 22 日由本保藏中心收到，并登记入册。根据您（们）的请求，由该日起保存三十年，在期满前收到提供培养物样品的请求后再延续保存五年。

该培养物的存活性本保藏中心于 2015 年 10 月 29 日检测完毕，结果为**存 活**。

中 国 典 型 培 养 物 保 藏 中 心

负责人（签名）[illegible]

2015 年 10 月 29 日
